# Impact of daily volumetric imaging on target tracking without fiducial markers during robotic treatment of pancreas

**DOI:** 10.1088/1361-6560/ae6229

**Published:** 2026-05-08

**Authors:** James L Bedford, Simeon Nill, Uwe Oelfke

**Affiliations:** Joint Department of Physics, The Institute of Cancer Research and The Royal Marsden NHS Foundation Trust, London SM2 5PT, United Kingdom

**Keywords:** robotic radiotherapy, image registration, motion model, pancreas, tumour tracking

## Abstract

*Objective.* Treatment of pancreas using Cyberknife is usually accomplished using radio-opaque fiducial markers that can be visualised on the orthogonal kilovoltage imaging system, but implantation of markers can lead to complications. This study therefore describes a method for pancreas tracking without fiducial markers and estimates the improvement in accuracy that can be achieved by additional daily pre-treatment volumetric imaging. *Approach.* Fiducial markers were artificially removed from the digitally reconstructed radiographs and treatment images of eight previously treated patients. Pancreas position was identified using a combination of local rigid registrations and inter- and intra-fraction motion models. The optimum effect of daily volumetric imaging was simulated by correcting the mean tracking position to match the mean position observed using fiducials. The impact of residual error on the dose distribution was estimated by shifting and recalculating the treatment plan. *Main results.* Median absolute tracking accuracy without fiducials over the 2823 acquired images is 3.8 mm (range 0.2–19.9 mm) without daily volumetric imaging. The lower bound of accuracy is 2.3 mm (0.1 mm—18.5 mm) when daily volumetric imaging is used. The former scenario reduces the *D*_95%_ of the planning target volume by a median of 2.5 Gy (1.0–10.2 Gy) compared to the planned dose distribution, whereas the latter scenario reduces *D*_95%_ by 1.4 Gy (0.4–2.8 Gy). In neither case is the *D*_95%_ of the clinical target volume (CTV) substantially lowered below the prescription. Median *D*_0.5cc_ to critical structures changes by less than 2 Gy. *Significance.* When tracking the target without fiducial markers, a planning target margin of 3–5 mm is sufficient to ensure that the CTV receives the desired prescribed dose, but the spatial accuracy and target coverage are considerably improved by using daily volumetric imaging.

## 1. Introduction

Stereotactic ablative body radiotherapy (SABR) is emerging is an effective treatment for cancers of the pancreas (Shouman *et al* 2024). However, accurate and effective radiotherapy of the pancreas is challenging due to the simultaneous difficulties of visualising the anatomy and accounting for considerable inter- and intra-fraction motion (Song *et al* 2015). Several studies have recently shown promise for improving accuracy by the use of an MR-guided approach (Stemkens *et al* 2015, Hall *et al* 2021, Grimbergen *et al* 2022a, 2022b), and 4D MRI also promises to allow for more accurate modelling of intra-fraction motion (Yang *et al* 2015, 2018 Deng *et al* 2017, 2019).

The Cyberknife robotic linear accelerator (Accuray Inc., Sunnyvale, CA) is ideally suited for the treatment of pancreatic tumours as it has a tracking subsystem which is valuable for real-time tracking of moving targets (Asmerom *et al* 2016, Ding *et al* 2018a, Kilby *et al* 2020). In this tracking subsystem, known as Synchrony, repeated orthogonal kilovoltage images are used in conjunction with optical tracking of a surface-based surrogate target to build a respiratory motion model. Tracking then proceeds on the surrogate system, with the model adjusted at regular intervals to match target position detected on periodic orthogonal images (Ferris *et al* 2020, Nano *et al* 2020). Although there are various systems available for accurate target tracking, all of which have their strengths (Colvill *et al* 2016), the Cyberknife system is the most sophisticated and flexible in terms of adjustment of the beam position and orientation to suit the presentation of target (Kumar *et al* 2025). The planning target volume (PTV) in this context is a moving volume designed to encompass the clinical target volume (CTV) with a sufficiently large margin that any errors in patient setup and tracking have negligible dosimetric impact on the CTV.

Visualisation of pancreas on the orthogonal images has hitherto required the use of implanted fiducial markers, but this may lead to surgical complications. There is therefore considerable motivation for tracking pancreas on the orthogonal kilovoltage images without the presence of fiducial markers. Deviations in position of the target volume from its position at the time of treatment planning can be quantified by comparing the measured orthogonal images (subsequently referred to as treatment images) with digitally reconstructed radiographs (DRRs) based on projections through the planning CT scan. Simple registration may be used to deduce the target position from the shift between treatment images and DRRs, although several more sophisticated methods have been reported. Zhao *et al* (2019) report on the use of deep learning to train a patient-specific network to deduce target locations from orthogonal images. They use DRRs based on deformed planning CT scans as a substitute for real images, which allows for more extensive training than might be possible with real images (Dhont *et al* 2020, Zhao *et al* 2021) but on the other hand, gives greater uniformity and consistency of data than is typically present in real patient images. Moreover, the approach uses a patient-specific model which requires fiducial markers for training, and it is therefore not suitable for true marker-less applications. A similar study is described by Zhou *et al* (2022) for the case of a gimballed linear accelerator, also using orthogonal kV images.

Nakao *et al* (2022) use a much more sophisticated deep learning method to track organ motion and deformation in the pancreas region. They use mesh structures to represent the most important structures in the abdomen. They then use a U-Net-based network to calculate a distribution of shift vectors over the 2D image planes. Points in the meshes are projected to the 2D image planes and the shift is looked up, and then a graph convolutional network is used to handle the deformation of the different organs, particularly the possibility that several different mesh points, all projecting to the same 2D image point, may have different deformations. The two networks operate together to give both shifts and deformations of the abdominal organs. The authors demonstrate the use of the network in a study of more than 150 patients with a mean absolute error in organ shape of approximately 5 mm, and 3.5 mm for the pancreatic gross tumour volume (GTV), although the study is also based on DRRs rather than real images.

The message from these prior studies of artificial intelligence (AI) is that, despite using DRR simulations which tend to make the task easier, the methods still have not performed very well, so at the moment, the conventional methods are still competitive. On the other hand, it is possible that the AI approaches will eventually take over this application (Mylonas *et al* 2021, Salari *et al* 2024). Also, AI can be used for image registration itself (Teuwen *et al* 2022), so the demarcation between image registration versus AI is not definite.

A further potential improvement in marker-less tracking accuracy can be made by the use of daily volumetric imaging such as a daily cone-beam CT scan. This permits the gross position of the pancreas to be determined before each fraction of treatment, so that the orthogonal kilovoltage images are only required to follow the respiratory and intra-fraction motion of the pancreas. This approach has been successfully implemented in a non-commercial context (Papalazarou *et al* 2017) and it is therefore desirable to understand more fully how it might benefit pancreas tracking without fiducials.

This study therefore presents a method which is specific to the Cyberknife geometry and which uses real patient images to perform marker-less tracking in pancreatic cancer patients. The method, which is based partly on image registration techniques and partly on a motion model, is first described, then the accuracy is evaluated in a cohort of previously treated pancreatic cancer patients. The optimum impact of daily volumetric imaging is then evaluated. The impact of residual tracking error, both with and without daily volumetric imaging, is estimated by recalculating and summing treatment plans for the observed errors.

## 2. Methods

### 2.1. Patients and clinical treatments

Eight patients were treated on the Cyberknife system at this centre for lesions in the head of the pancreas since 2013 and all of these patients were retrospectively studied. For the treatment, standard departmental protocols were followed. Four gold seed fiducial markers were surgically implanted into the head of the pancreas and the patients were CT scanned with 1 mm slice spacing. Patients 1–7 were scanned with 4DCT and the average scan was used for planning, but patient 8 was the most historical and was scanned helically. For most of the cases the CTV was equal to the GTV. The fiducial markers were identified on the CT scan within the Precision treatment planning system (Accuray) and if the positioning and separation of the markers was adequate for tracking both shifts and rotations, a 3 mm PTV margin was created. In the event of fiducial markers overlapping in the plane of the orthogonal imaging system or being misplaced with respect to the target volume, a 5 mm margin was used. Due to the nature of the surgical procedure, some of the fiducial markers were often located outside of the target volume. Treatment plans were then created in Precision for a prescribed dose of 30–45 Gy to approximately 80% of the maximum dose, such that 95% of the PTV was covered, in 3–5 fractions, and DRRs were constructed. Table 1 summarises the treatment of each patient and also shows the range of intra-fraction motion encountered in each patient.

**Table 1.** Patient information.

Patient	Dose (Gy)	Fractions	PTV margin (mm)	Fiducials in PTV^a^	*L*/*R* amp (mm)^b^	*A*/*P* amp (mm)^b^	*S*/*I* amp (mm)^b^
1	35	5	5	1	7.1	9.3	13.9
2	36	3	3	3	7.6	8.6	15.4
3	45	5	5	1	11.4	15.4	23.8
4	30	5	5	3	19.3	21.7	19.8
5	30	5	5	3	19.2	7.8	26.6
6	35	5	3	1 (+stent)	17.0	9.7	18.4
7	35	5	5	1	7.4	7.5	17.8
8	38	3	3	0	27.8	11.2	34.1

^a^ Number of fiducials located in the PTV itself, excluding those in the vicinity.

^b^ Peak–peak amplitude of intra-fraction fiducial motion. *L*: left, *R*: right, *A*: anterior, *P*: posterior, *S*: superior, *I*: inferior.

During treatment delivery, Synchrony tracking was used (Nano *et al* 2020), with an external abdominal marker being continuously monitored and related to internal motion by a motion model. The motion model was constructed at the start of each fraction by correlating target position and orientation as determined from repeated orthogonal images with the position of the external surrogate. The applicability of the motion model was verified at intervals during the treatment by acquisition of repeat kV images at energy between 110 kV and 135 kV.

### 2.2. Retrospective study

For the retrospective analysis described in this paper, the Data Extractor Tool (Accuray, Inc.), normally used for sending patient data to Accuray for analysis, was used to export a file structure to disk, with this file structure containing the treatment images and tracking information relating to the patient treatment. In-house software CKImage v1.3 was then used to interrogate the file structure and to present the images, the actual tracked positions during the treatment, and the tracked positions obtained without fiducials. CKImage was based upon the AutoBeam in-house treatment planning framework, using many of its data structures (Bedford 2024). The software was written in multithreaded Java (Oracle Corporation, Reading, UK) for speed and portability.

In the eight patients, a total of 2826 pairs of treatment images were considered. Three pairs of images from patient 8 were removed from the analysis as upon inspection of the images, the reported tracking positions during treatment were more than 20 mm away from the actual fiducial positions, indicating a difficulty in registering the fiducial markers at the time of treatment. The remaining 2823 images were analysed.

**Figure 1.**
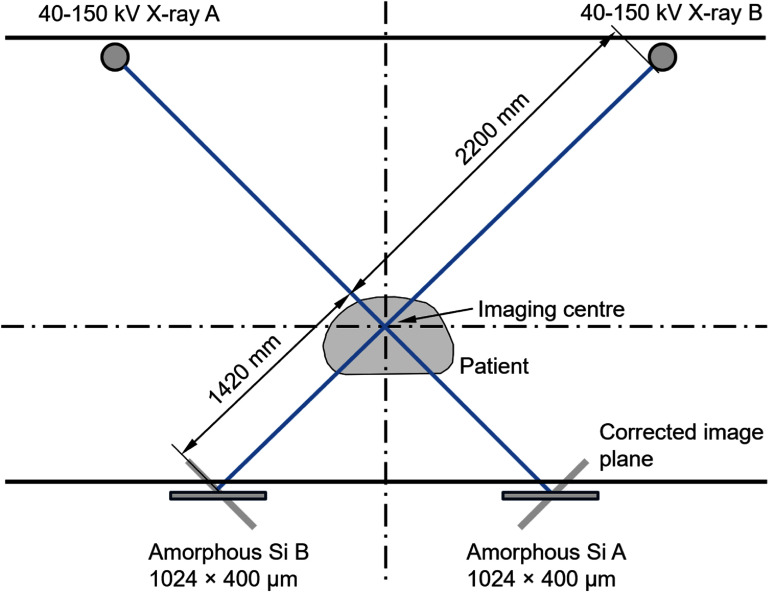
Imaging geometry of the Cyberknife system.

DRRs for registration of the treatment images in the retrospective study were generated internally using CKImage, using the average scan from the 4DCT in patients 1–7 and the helical scan in patient 8. The process used ray tracing with a beam energy of 120 kV and an attenuation coefficient of 0.0171 mm^−1^ according to ICRU 46 (ICRU 1992). During this process, a modified CT-to-density table was used, so that regions of the CT scan with a mass density higher than 1.869 g cm^−3^ were assigned to a value of 1.0 g cm^−3^. This was a convenient means of hiding the fiducial markers. The imaging geometry is shown in figure 1.

### 2.3. Image-based target tracking

**Figure 2.**
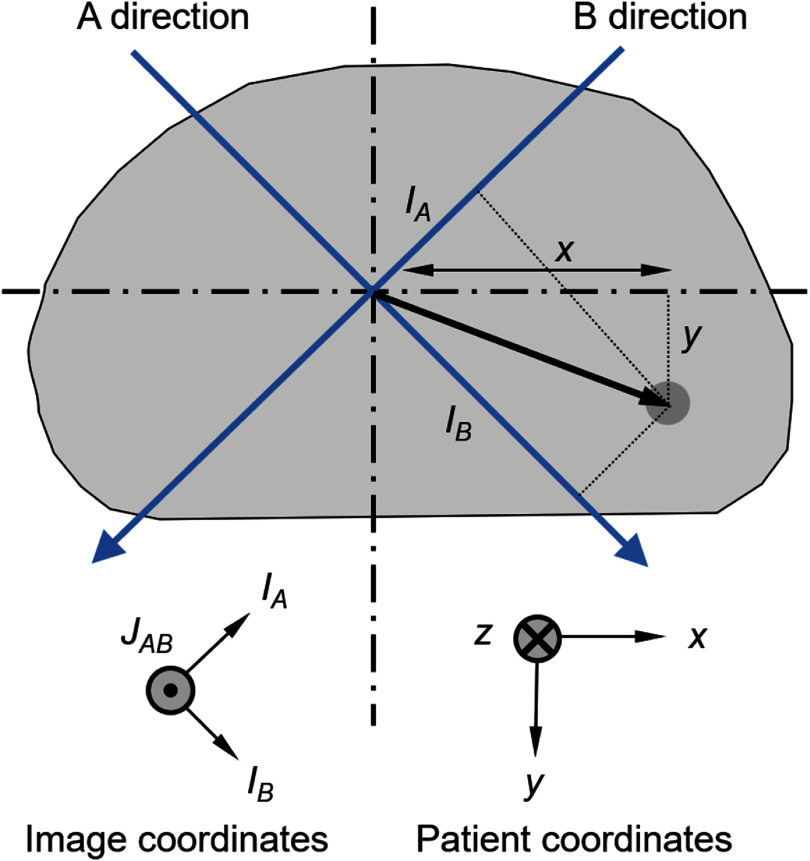
Relationship between two-dimensional image projections and three-dimensional target position.

The approach used in this study was to register the treatment images to the DRRs to determine the pancreatic shift. A superior–inferior shift of the pancreatic region of the treatment images with respect to the DRRs was expected to manifest itself in both *A* and *B* images simultaneously due to the geometry shown in figure 1, whereas left–right and anterior–posterior shifts were expected to result in independent shifts in the *A* and *B* images. For left–right shifts of *I_A_* and *I_B_* in the *A* and *B* images, respectively, and a superior–inferior shift of *J_AB_* in the two images simultaneously, the resulting shift (*x, y, z*) in the pancreatic volume of interest (figure 2) was:

\begin{equation*}x = R\left( {{I_A} + {I_B}} \right),\end{equation*}

\begin{equation*}y = R\left( {{I_B} - {I_A}} \right),\end{equation*}

\begin{equation*}z = - {J_{AB}},\end{equation*}

where $R = {{\sqrt 2 } \mathord{\left/ {\vphantom {{\sqrt 2 } 2}} \right. } 2}$.

One could therefore envisage taking the central part of the treatment images, registering them to the DRRs and obtaining the desired shift. However, this was not used in practice as the treatment images contained various anatomical elements which were not fixed in relation to each other. Most notably, the spine overlaid the pancreatic region and was reasonably rigid, although varied slightly in position during treatment as the patient was not completely immobilised. The pancreatic region itself was subject to bowel movements, and the diaphragm was located superiorly in the images, which moved with respiration. In addition, accurately registering the central part of the images was difficult due to the limited information in the treatment images, so the relationship given above in equations (1) was not used directly. Instead, a series of registrations was performed on a 5 × 5 matrix of subregions. The result was a 5 × 5 grid of shift vectors for each of the two treatment images, giving the shift of each part of those images with respect to the DRRs. A further special image registration was carried out for the rectangular region encompassing the beam’s eye view of the PTV, plus a 20 mm margin. This was to give specific emphasis to the region of the image in which the pancreatic target volume lay. The registration vectors were then applied to a motion model which transformed them to pancreatic shift vectors. Note that the motion model could potentially provide the simple linear relationship described in equations (1) if this was desirable, but the model also had the scope to provide other linear combinations of the registration components as well and was therefore more general. Further details of these steps are given the following sections.

### 2.4. Image registration

For tracking purposes, processed Cyberknife images were used. These were of dimensions 320 × 320 pixels, corrected to an image plane orthogonal to the x-ray beam axis (figure 1). Each of these images was further filtered to improve the spatial resolution. A bandpass filter was used for this purpose, with magnitude $1 + gf$ from 0 to 0.5 *f*_max_ followed by magnitude $1 + g\left( {f - {f_{{\mathrm{max}}}}} \right)$ from 0.5 *f*_max_ to *f*_max_, where *f*_max_ was the Nyquist frequency and *g* was the filter gradient, equal to 0.02. This filter gradient was chosen empirically to improve the visual sharpness of the images, without producing excessive high-frequency speckle.

The fiducial markers were then removed from the images. This was accomplished by selecting the 0.5% of pixels with the highest intensity in the central region spanning from 0.25 to 0.75 of the image width and length. Each pixel in that selection, together with its immediate neighbours was then assigned a value equal to the mean of the intensities of the pixels located 10 pixels left, right, above and below that pixel. In other words, the fiducials were replaced with mean intensity of the surrounding pixels. There was a possibility that this could introduce a bias into the images, or to leave a trace that could assist the tracking algorithm in following the pancreas, particularly where the pancreas overlapped with bony anatomy or regions of low density. However, this was not considered to be an issue in practice as the fiducial markers were very well obscured by the replacement method.

The DRR images were then equalised in intensity to the treatment images. The image region was divided into a grid of 5 × 5 smaller regions. The 25^th^ and 75^th^ percentiles of both DRR image and treatment image intensity were then found for each of the four corner regions of this grid. Bilinear interpolation was then used to calculate these percentiles for all pixels in the image. Each individual pixel was then assigned a new intensity, $D_{ij}^{\mathrm{eq}}$:

\begin{equation*}D_{ij}^{\mathrm{eq}} = \left( {{D_{ij}} - {d_{\min }}} \right)s + {t_{\min }},\end{equation*}

where ${D_{ij}}$ was the original intensity, *d*_min_ was the interpolated 25^th^ percentile image value of the DRR image and *t*_min_ was the corresponding 25^th^ percentile image value of the treatment image. The factor *s* was the rescale factor:

\begin{equation*}s = \left( {{t_{{\mathrm{max}}}} - {t_{{\mathrm{min}}}}} \right)/\left( {{d_{{\mathrm{max}}}} - {d_{{\mathrm{min}}}}} \right),\end{equation*}

where *t*_max_ was the interpolated 75^th^ percentile of the treatment image and *d*_max_ was the 75^th^ percentile of the DRR image. Effectively, this method scaled the interquartile range of image intensities in the DRR images to match that of the treatment images, but in a smoothly varying manner due to the use of bilinear interpolation to control the correction factors.

Image registration itself consisted of locating corresponding regions on DRRs and treatment images by shifting the treatment images with respect to the DRRs and evaluating an objective function to assess the quality of each shift:

\begin{equation*}\gamma \left( {I,J} \right) = \sum\limits_{ij} {f\left\{ {T\left( {i + I,j + J} \right)-D\left( {i,j} \right)} \right\}} ,\end{equation*}

where *T*(*i, j*) referred to the treatment image and *D*(*i, j*) referred to the DRR image, *i* and *j* referred to the indices of the image pixels and *I* and *J* represented the shifts in the left–right and superior–inferior directions, respectively. The first step of image registration used the *A* and *B* images in a coupled manner due to the simultaneous manifestation of superior–inferior shifts in both *A* and *B* images. A nested optimisation routine was used to apply various superior–inferior image shifts, *J_AB_* to both *A* and *B* images simultaneously and for each of these shifts, separate sideways shifts, *I_A_* and *I_B_*, were applied to the *A* and *B* images respectively:

\begin{equation*}\gamma \left( {{I_A},{I_B},{J_{AB}}} \right) = \sum\limits_{ij} {f\left( {{T_A}\left( {i + {I_A},j + {J_{AB}}} \right)-{D_A}\left( {i,j} \right)} \right)} + \sum\limits_{ij} {f\left( {{T_B}\left( {i + {I_B},j + {J_{AB}}} \right)-{D_B}\left( {i,j} \right)} \right)} \end{equation*}

where the subscripts *A* and *B* referred to the *A* and *B* images. Two passes of this process were performed, the first using a large range (±20 pixels) and coarse step size (4 pixels) and the second using a small range (±2 pixels) and fine step size (1 pixel). The entire image was used for this process.

The resulting overall image shift was used as the starting value for a further region-specific registration in each of the 5 × 5 subregions, each region overlapping with the adjacent regions by 50% to encourage robustness and uniformity of result. Two passes of this registration were performed, the first using a large range (±10 pixels) and coarse step size (2 pixels) and the second using a small range (±1 pixels) and fine step size (1 pixel).

The objective function used during this process was the product of a normalised mean square (NMS) objective and a normalised mutual information (NMI) objective. The rationale for this was that the NMS objective gave the mapping of spatial information, whereas the NMI objective gave additional robustness where the image intensities of DRR and treatment images were not fully matched. Efforts were made to match the intensity of these images (equation (2)), but some differences inevitably remained. The use of NMI was designed to overcome remaining differences in intensity, being based on information content rather than spatial features (Studholme *et al* 1999, Czolbe *et al* 2023). The value of adding NMI to NMS was verified empirically to improve robustness of registration.

The NMS objective function was given by:

\begin{equation*}\operatorname{NMS} = 1-\frac{1}{{Nn}}{\sum\limits_{ij} {\left( {T\left( {i + I,j + J} \right)-D\left( {i,j} \right)} \right)} ^2}\end{equation*}

where *n* was the number of pixels in the region being registered and *N* was the normalisation factor, given as:

\begin{equation*}N = {\left[ {\max \left( {{D_{\max }} - {T_{\min }},\;{T_{\max }} - {D_{\min }}} \right)} \right]^2}\end{equation*}

representing the largest magnitude of mean square that could be encountered. The subscripts *A* and *B* have been dropped.

The NMI was calculated from the entropy, $\Psi $, in the images:

\begin{equation*}\operatorname{NMI} = {{\left( {\Psi \left( {\mathbf{D}} \right) + \Psi \left( {\mathbf{T}} \right){\text{ }}} \right)} \mathord{\left/ {\vphantom {{\left( {\Psi \left( {\mathbf{D}} \right) + \Psi \left( {\mathbf{T}} \right){\text{ }}} \right)} {\Psi \left( {\mathbf{M}} \right)}}} \right. } {\Psi \left( {\mathbf{M}} \right)}}\end{equation*}

where **D** and **T** were histograms of intensity in the DRR and treatment images, respectively and **M** was the joint histogram for both DRR and treatment images (Wang 2014). The histograms consisted of 128 bins of equal width covering the range of intensities in the region being registered, with the lower limit of each bin denoted by *L*. Then **M** represented the probability of finding a given intensity *L_d_* (*d* = 0…127) in the DRR and a given intensity, *L_t_* (*t* = 0…127) in the treatment image:

\begin{equation*}{{\mathbf{M}}_{dt}} = P\left( {{L_d} \unicode{x2A7D} {D_{ij}} &lt; {L_{d + 1}}{\text{ and }}{{\mathrm{L}}_t} \unicode{x2A7D} {T_{i + I,j + J}} &lt; {L_{t + 1}}} \right).\end{equation*}

Note that **D** was the row-wise sum of **M**, while **T** was the column-wise sum. The entropy itself was calculated as:

\begin{equation*}\Psi = - \sum\limits_{dt} {\left( {{\mathbf{M}}{}_{dt}\ln {\mathbf{M}}{}_{dt}} \right)} .\end{equation*}

The total objective value was then:

\begin{equation*}\gamma = {\mathrm{NMS}} \times {\mathrm{NMI}}.\end{equation*}

This was used in equation (5).

For the pancreas-specific registration within the PTV region, a coupled registration was used according to equation (5) on the rectangle encompassing the PTV plus a 20 mm margin, but then no further adjustment was made. In summary, the image registration step consisted of registering both the complete images in a quasi-deformable approach, plus the pancreas-specific region in a coupled, rigid approach.

### 2.5. Motion model

**Figure 3.**
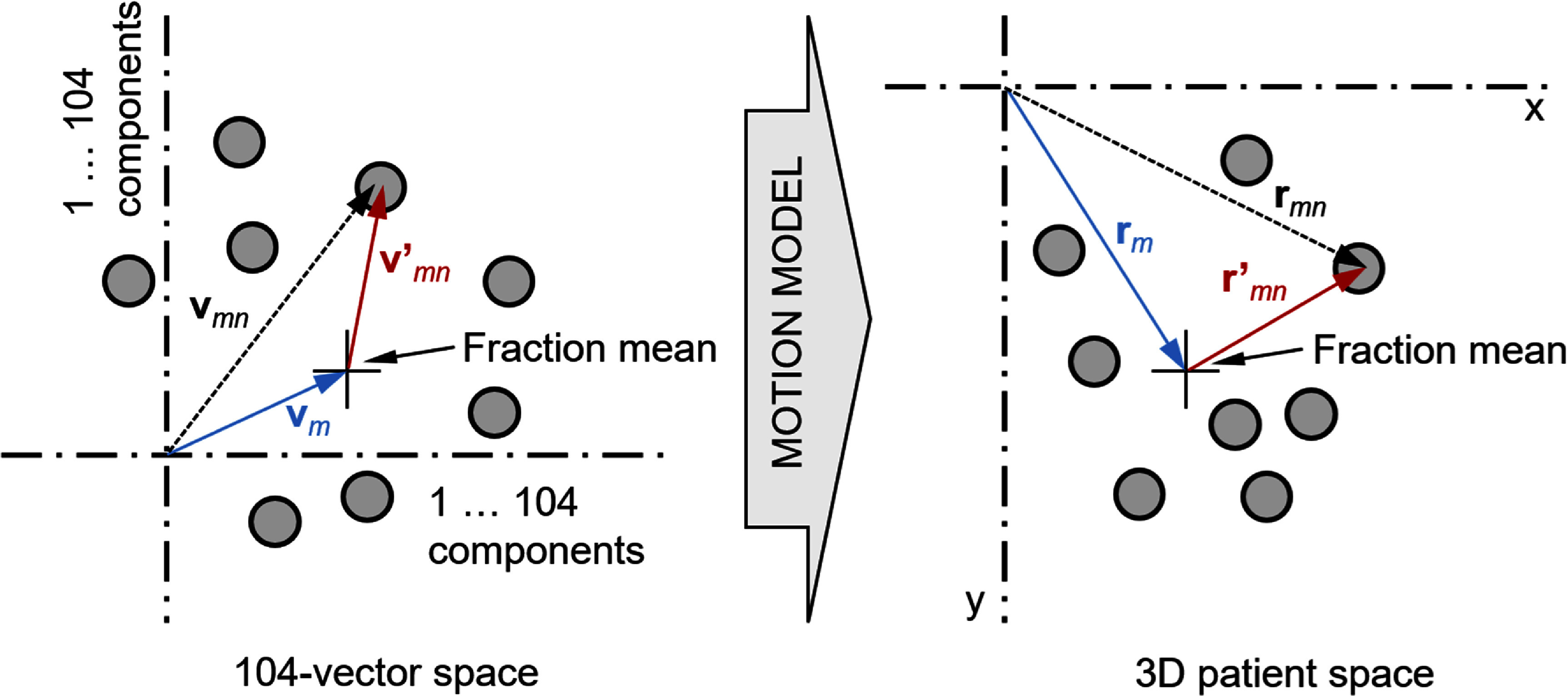
Motion model.

The result of the above image registration was a set of 2 × 26 registration vectors (2 images, each with 25 grid regions plus 1 specific PTV region). Each of the vectors consisted of two components: an antero-lateral shift and a supero-inferior shift, giving 104 components. These 104 components were considered as a 104-vector representing the motion state in image space, and the motion model then mapped this vector into a three-dimensional vector giving the corresponding position of the target volume. The motion model was divided into inter-fraction and intra-fraction components:

\begin{equation*}{{\mathbf{v}}_{mn}} = {{\mathbf{v}}_m} + {{\mathbf{v^{\prime}}}_{mn}}\quad \Rightarrow \quad {{\mathbf{r}}_{mn}} = {{\mathbf{r}}_m} + {{\mathbf{r^{\prime}}}_{mn}},\end{equation*}

where **v***_mn_* represented the 104 image components at fraction *m* (*m* = 1…*M*) and image instance *n* within that fraction (*n* = 1 … *N_m_*), **v***_m_* was the corresponding set of image components representing the state at fraction *m* as a whole, and ${{\mathbf{v^{\prime}}}_{mn}}$ was the set of intra-fraction image components. In patient space, **r***_mn_* was the position of the target volume at fraction *m* and image instance *n* within that fraction, **r***_m_* was the inter-fraction target position at fraction *m*, and ${{\mathbf{r}}_{mn}}^{\prime} $ was the intra-fraction position at fraction *m* and image instance *n* (figure 3). Without loss of generality, **v***_m_* and **r***_m_* were taken to be the mean of values **v***_mn_* and **r***_mn_*, respectively, over each fraction.

Then:

\begin{equation*}{{\mathbf{r}}_m} = {f_{inter}}\left( {{{\mathbf{v}}_m}} \right)\end{equation*}

\begin{equation*}{{\mathbf{r^{\prime}}}_{mn}} = {f_{intra}}\left( {{{{\mathbf{v^{\prime}}}}_{mn}}} \right)\end{equation*}

so that:

\begin{equation*}{{\mathbf{r}}_{mn}} = {f_{inter}}\left( {{{\mathbf{v}}_m}} \right) + {f_{intra}}\left( {{{{\mathbf{v^{\prime}}}}_{mn}}} \right).\end{equation*}

Each of the two models was then expressed as:

\begin{equation*}{\mathbf{Ax}} = {\mathbf{b}}\end{equation*}

where *A_ij_* consisted of the *i*th observation (*i* = 0…2822) of the *j*th registration vector (*j* = 0…103) and *A_i_*_104_ = 1. The values of *b_ij_* were the *i*th observation of the *j*th 3D vector component of the pancreatic shift (i.e. *j* = 0 represented *x, j* = 1 represented *y* and *j* = 2 represented *z*). This was then solved using singular value decomposition (Press *et al* 1989):

\begin{equation*}{\mathbf{A = U}}\;{\mathbf{W}}\;{{\mathbf{V}}^T}\end{equation*}

where **U** and **V** were orthogonal matrices:

\begin{equation*}{{\mathbf{U}}^T}\,{\mathbf{U}} = {{\mathbf{V}}^T}\,{\mathbf{V}} = 1\end{equation*}

and **W** was a diagonal matrix of singular values. The columns of **U** formed a basis for the range of **A** and the columns of **V** formed a basis for the null space of **A**, in a similar approach to principal components analysis (Fransson *et al* 2021). Then in principle:

\begin{equation*}{\mathbf{x = V}}\,{{\mathbf{W}}^{ - 1}}\,{{\mathbf{U}}^T}\,{\mathbf{b}}.\end{equation*}

However, to regularise the solution, values of **W**^−1^ less than *W*_max_/*R* were set to zero before application of equation (18), as these represented vector components making a negligible contribution to the quality of the linear fit or were contributing to over-fitting. The value of *R* was determined empirically using a simple grid search and was set to 1.2 for the inter-fraction model and 7.0 for the intra-fraction model.

To ensure that overfitting was not occurring, leave-one-out cross validation was used. Fitting was performed for seven of the eight patients and the model was validated on the eighth patient. All eight patients were used in turn as the validation patient, thereby providing eight results.

### 2.6. Daily volumetric imaging

To assess the impact of daily volumetric imaging, such as cone-beam CT, on the tracking accuracy, two scenarios were modelled during validation on each of the patients. The first scenario assumed no prior knowledge as to the position of the PTV, so that the inter-fraction model was used to predict the mean position for each fraction. The second scenario assumed that pre-fraction daily volumetric imaging could be used to determine the mean position of the PTV at each fraction. Consequently, equation (14) could be modified to:

\begin{equation*}{{\mathbf{r}}_{mn}} = {{\mathbf{r}}_m} + {f_{intra}}\left( {{{{\mathbf{v^{\prime}}}}_{mn}}} \right)\end{equation*}

so that the motion model was used to predict changes from the mean position only. Note that this step involved the assumption that the mean position could be accurately determined from the daily volumetric imaging.

### 2.7. Dosimetric validation

Retrospective treatment plans were produced for the eight patients in AutoBeam v6.4 in-house treatment planning software using a beam model representing the 6 MV flattening filter-free beam of the Cyberknife system. The standard body path of the Cyberknife robot was used to define robot positions, and the S7 multileaf collimator with leaf width of 3.85 mm at 800 mm source-axis distance was also modelled. The prescribed dose was taken to be 35 Gy in 5 fractions for all cases, except for patient 4, who was planned to 30 Gy in 5 fractions so as to respect the dose-limiting constraints of the stomach and small bowel (as also with the original clinical plan). The normal tissue constraints were taken from the UK SABR guidelines (RCR 2019). The PTV margin used for these treatment plans was the same as that used for clinical treatment (see table 1). Dose was calculated by a fast convolution algorithm (Bedford 2002) on a grid of 2 mm × 2 mm × 2 mm resolution covering the entire patient CT scan.

The target positions calculated by the motion model were compared with the positions determined by the Cyberknife system using fiducial markers. For this comparison, only the shift component of the Cyberknife tracking position was used as the rotational component was not used clinically for many of the cases in the study.

The dosimetric impact of the residual difference after tracking with CKImage was determined by shifting the treatment plan accordingly. For each patient, the initial treatment plan was repositioned with an offset equal to the residual shift. The stochastic nature of tracking, in which a different residual shift occurred during delivery of each beam of the plan (Bedford *et al* 2020), was modelled by randomly selecting the index of the residual shift to be applied to each beam. A uniform distribution with width equal to the number of available images for the given patient was used for this purpose. This process was repeated for the five treatment fractions and the dose for all fractions accumulated to assess the dose after the entire treatment course. This was repeated 10 times and the range of dose-volume histograms was calculated. The expectation value of the dose distribution was also calculated by assuming an infinite number of shifts, so that all parts of the treatment plan received all shifts. The calculation therefore applied all available residual shifts to the entire treatment plan. This process required around 12 h of computation time on a 128-thread AMD Ryzen Threadripper 3.2 GHz processor (AMD, Santa Clara, CA).

## 3. Results

### 3.1. Tracking accuracy without daily volumetric imaging

**Figure 4.**
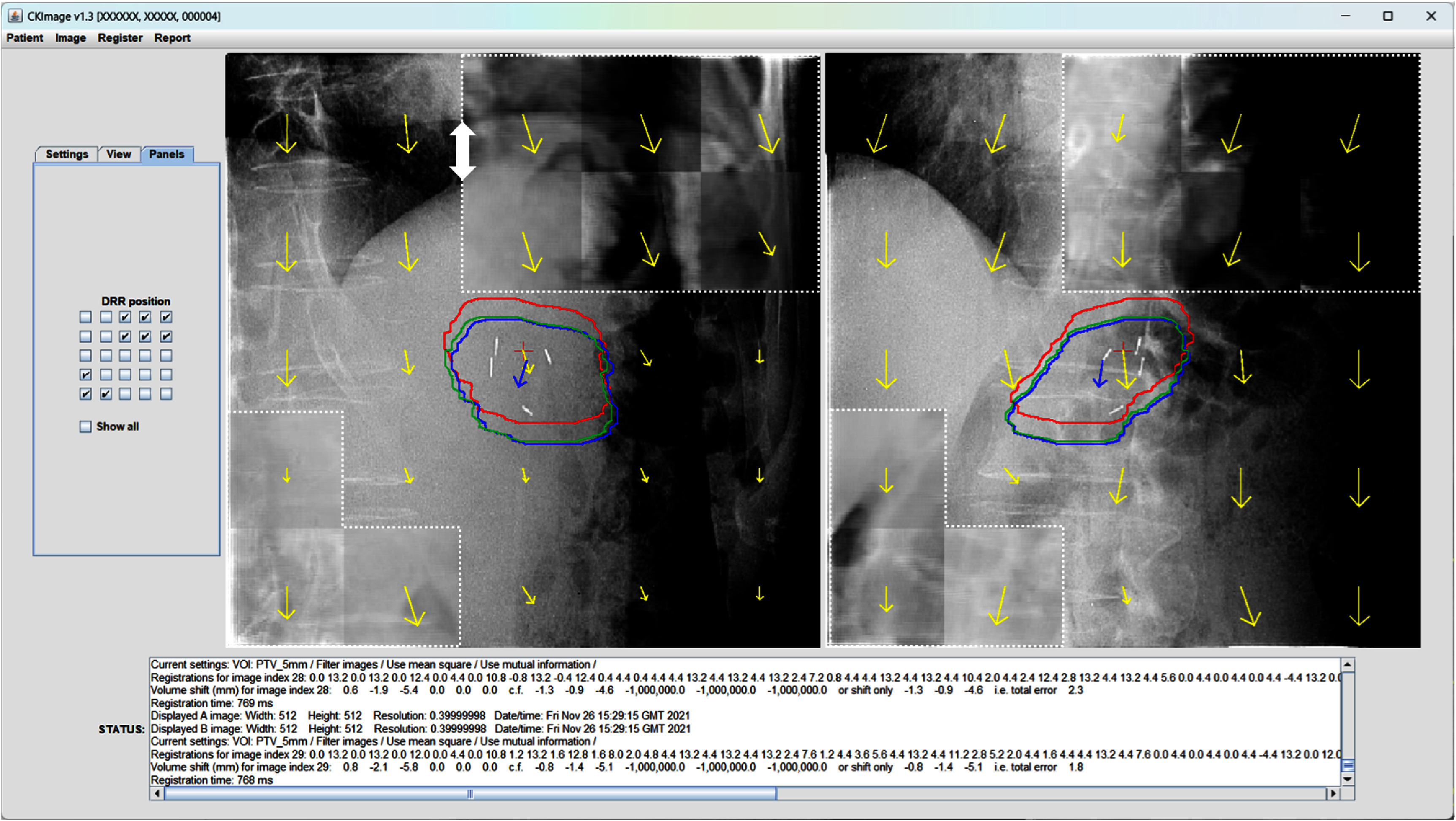
CKImage v1.3. The two images are the two orthogonal kV Cyberknife images. The corresponding digitally reconstructed radiographs (DRRs) are exposed for illustrative purposes in the top right and bottom left of each image. The difference in diaphragm position between the DRR and the treatment image is highlighted at the top of the left image by the white arrow. The yellow arrows show the registration of the treatment images with the DRRs (downwards in this example because the diaphragm is positioned downwards). The fiducials are visible at the centre, but these are removed by image processing for analysis purposes. The red area represents the PTV projected from the planning CT scan, therefore registered inherently with the DRR, the blue area represents the PTV tracked without fiducials using a motion model, and the green area represents the position of the PTV as tracked with fiducials by the Cyberknife system at the time of treatment.

Typical appearance of the tracking software is shown in figure 4. This figure illustrates the registration regions, the vector components and the target locations derived from the vector components. Once the patient and DRRs are loaded into the tracking software, the actual registration and application of the motion model to produce the results shown in the figure takes around 0.8 s.

**Figure 5.**
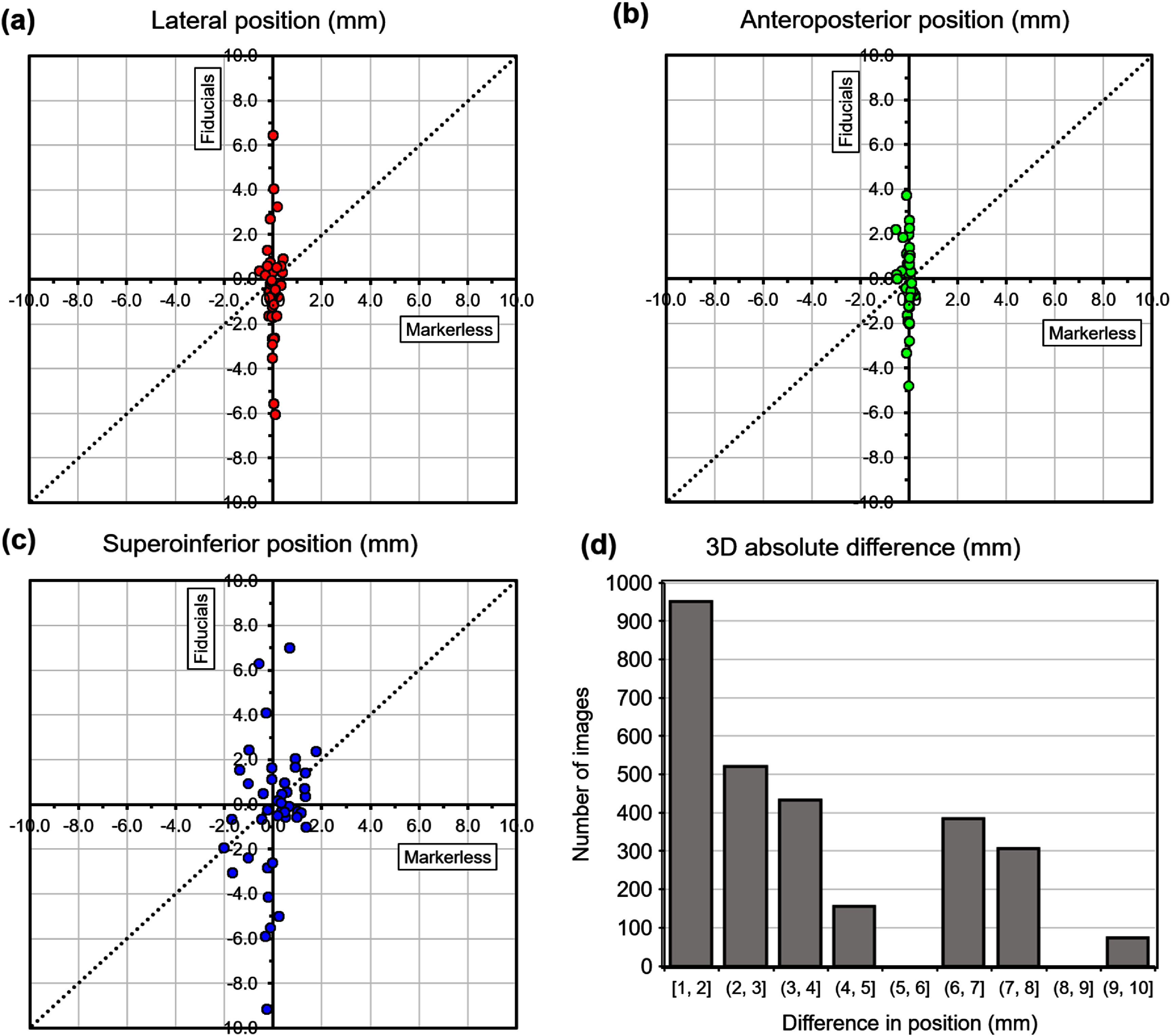
**Inter-fraction** pancreas position predicted by marker-less tracking, compared to tracking with fiducials, in (a)–(c) three orthogonal directions, and (d) all directions combined, **without** daily volumetric imaging.

**Figure 6.**
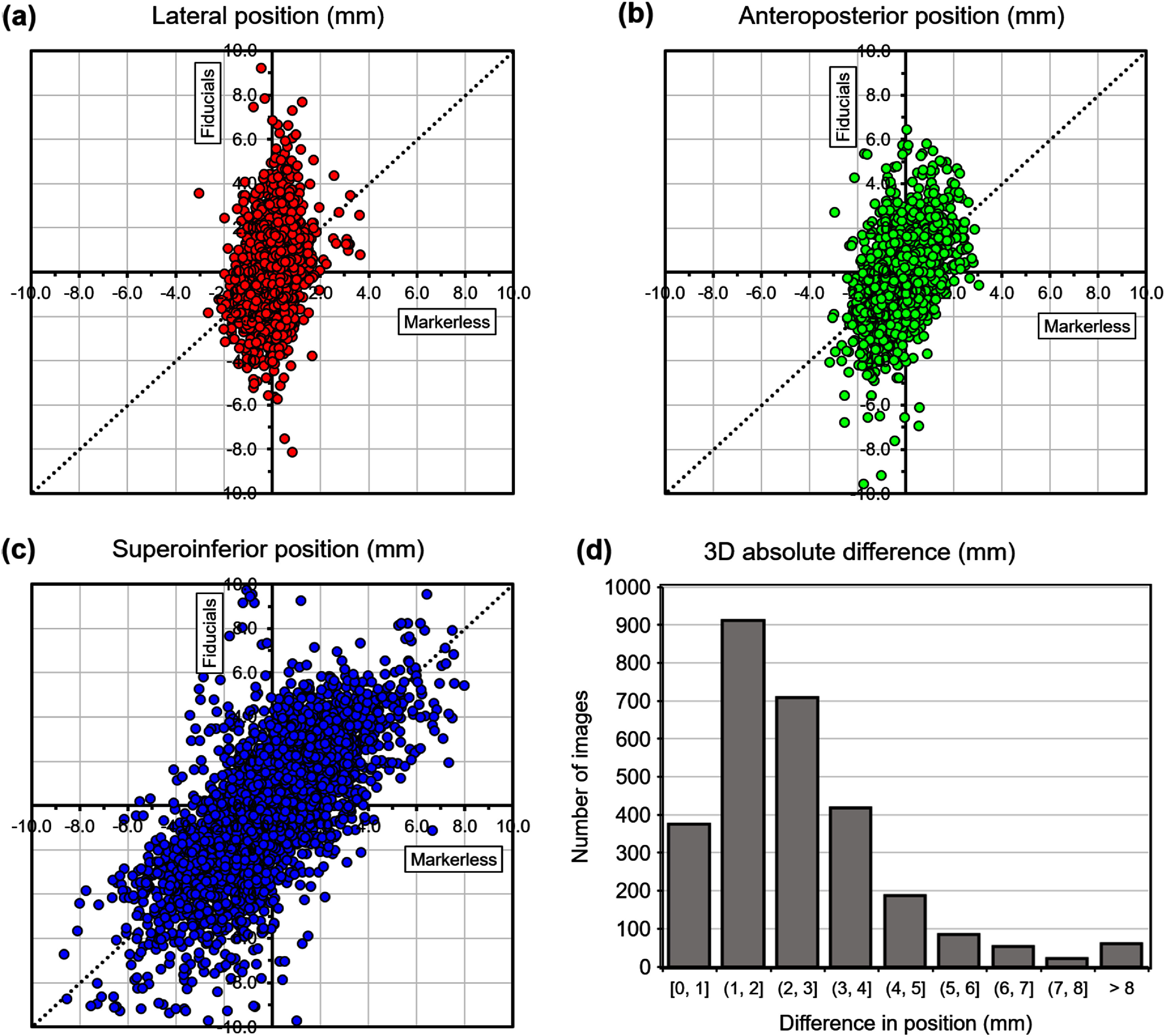
**Intra-fraction** pancreas position predicted by marker-less tracking, compared to tracking with fiducials, in (a)–(c) three orthogonal directions, and (d) all directions combined, **without** daily volumetric imaging.

The accuracy of model fitting is given in table 2, where the evaluation is for the same patient subsets that are used for fitting the model. Table 3 then gives the accuracy of the model as applied to the test patients in the leave-one-out cross validation. Figure 5 shows the results of the inter-fraction model applied to the test patients, relating the mean registration vector values for each fraction to the mean three-dimensional target position vector for each fraction. Figure 6 then shows the accuracy of the intra-fraction motion model on the test patients. Table 4 shows the median accuracy of the model in the three orthogonal directions.

**Table 2.** Tracking errors on the patients used for **fitting** of the motion model, **without** daily volumetric imaging. Key statistics are given in bold.

Patients	Minimum (mm)	Lower quartile (mm)	Median (mm)	Upper quartile (mm)	Maximum (mm)
1–8 excluding 1	0.1	2.4	**4.0**	6.1	18.9
1–8 excluding 2	0.3	2.3	**3.9**	6.2	19.3
1–8 excluding 3	0.2	2.1	**3.5**	6.1	19.6
1–8 excluding 4	0.2	2.3	**3.7**	6.0	20.1
1–8 excluding 5	0.2	2.2	**3.4**	5.3	20.2
1–8 excluding 6	0.1	2.0	**3.2**	5.6	19.0
1–8 excluding 7	0.2	2.4	**4.0**	6.3	19.6
1–8 excluding 8	0.0	2.1	**3.4**	5.5	15.8

**Table 3.** Tracking errors on the patients used for **testing** of the motion model, **without** daily volumetric imaging. Key statistics are given in bold.

Patient	Minimum (mm)	Lower quartile (mm)	Median (mm)	Upper quartile (mm)	Maximum (mm)
1	0.4	1.5	**2.1**	3.0	8.5
2	0.2	1.7	**2.2**	2.9	8.0
3	0.2	2.6	**3.8**	5.4	16.4
4	0.4	2.6	**4.1**	5.7	15.4
5	0.4	3.3	**5.9**	8.0	12.6
6	0.5	3.6	**5.1**	6.8	15.2
7	0.2	1.5	**2.4**	3.3	8.1
8	1.3	4.5	**6.5**	8.9	19.9
All patients	**0.2**	**2.3**	**3.8**	**6.1**	**19.9**

**Table 4.** Directional components of tracking errors on the patients used for testing of the motion model, **without** daily volumetric imaging. Key statistics are given in bold.

Direction	Minimum (mm)	Lower quartile (mm)	Median (mm)	Upper quartile (mm)	Maximum (mm)
Left–right	0.0	0.7	**1.6**	3.2	12.9
Antero-posterior	0.0	0.6	**1.3**	2.3	11.1
Supero-inferior	0.0	0.9	**2.1**	4.0	14.7
All directions	**0.2**	**2.3**	**3.8**	**6.1**	**19.9**

### 3.2. Tracking accuracy with daily volumetric imaging

**Figure 7.**
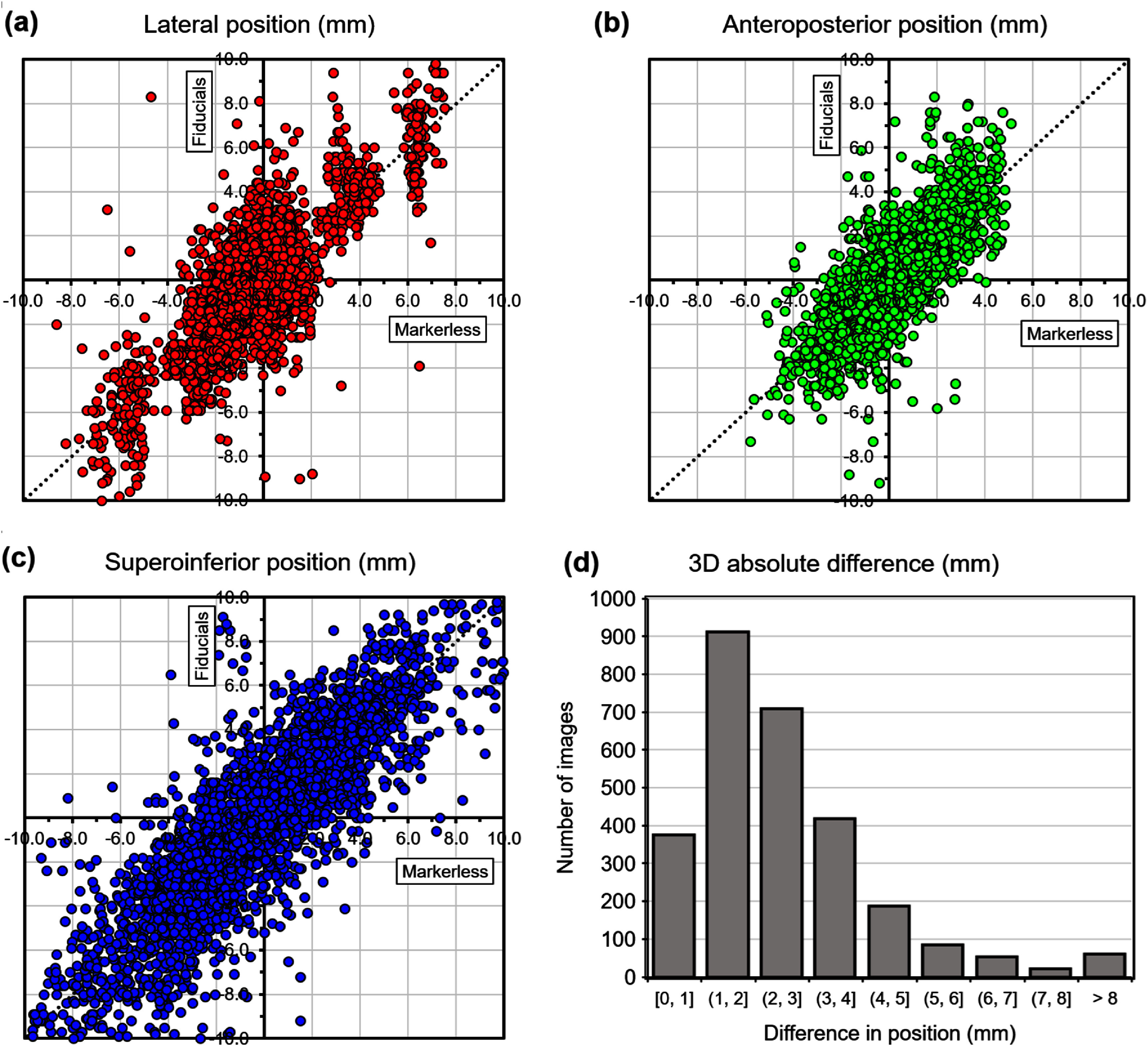
Pancreas position predicted by marker-less tracking, compared to tracking with fiducials, in (a)–(c) three orthogonal directions, and (d) all directions combined, **with** daily volumetric imaging.

Table 5 and figure 7 show the results of the model applied to the test patients when daily volumetric imaging is assumed. In other words, the inter-fraction mean value of the target position is taken directly from the test patients, rather than being predicted by the motion model derived from the fitting patients. Table 6 shows the accuracy of the directional components.

**Table 5.** Tracking errors on the patients used for **testing** of the motion model, **with** daily volumetric imaging. Key statistics are given in bold.

Patient	Minimum (mm)	Lower quartile (mm)	Median (mm)	Upper quartile (mm)	Maximum (mm)
1	0.2	1.1	**1.5**	1.9	8.2
2	0.2	1.1	**1.6**	2.1	7.9
3	0.3	2.0	**2.9**	4.0	11.2
4	0.4	2.1	**3.0**	4.1	16.0
5	0.1	1.5	**2.0**	2.9	13.5
6	0.4	1.8	**2.6**	3.5	13.9
7	0.2	1.1	**1.6**	2.4	8.4
8	0.2	1.9	**2.8**	4.5	18.5
All patients	**0.1**	**1.5**	**2.3**	**3.3**	**18.5**

**Table 6.** Directional components of tracking errors on the patients used for testing of the motion model, with daily volumetric imaging. Key statistics are given in bold.

Direction	Minimum (mm)	Lower quartile (mm)	Median (mm)	Upper quartile (mm)	Maximum (mm)
Left–right	0.0	0.4	**0.9**	1.7	14.7
Antero-posterior	0.0	0.4	**0.8**	1.4	13.2
Supero-inferior	0.0	0.6	**1.3**	2.4	15.4
All directions	**0.1**	**1.5**	**2.3**	**3.3**	**18.5**

### 3.3. Dosimetric accuracy

**Figure 8.**
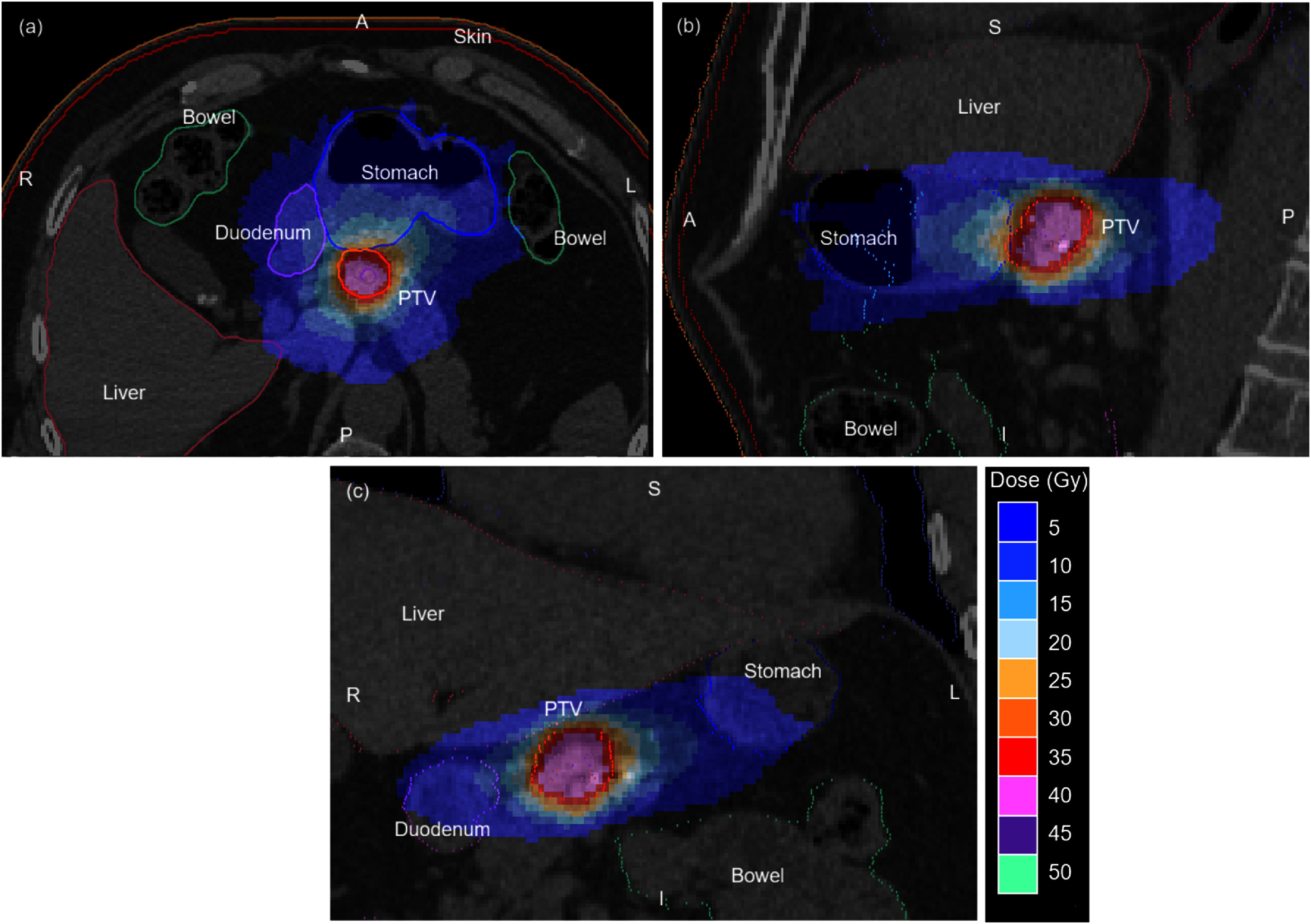
(a) Transaxial, (b) sagittal and (c) coronal views of a typical pancreatic target volume and treatment plan as used in the study of dosimetric impact of residual errors. *S*: superior, *I*: inferior, *L*: left, *R*: right, *A*: anterior, *P*: posterior.

A typical treatment plan is shown in figure 8. The dose distribution is slightly anteriorly weighted due to the use of predominantly anterior robot positions in the standard body path. The stomach, bowel and duodenum are the dose-limiting critical structures.

**Figure 9.**
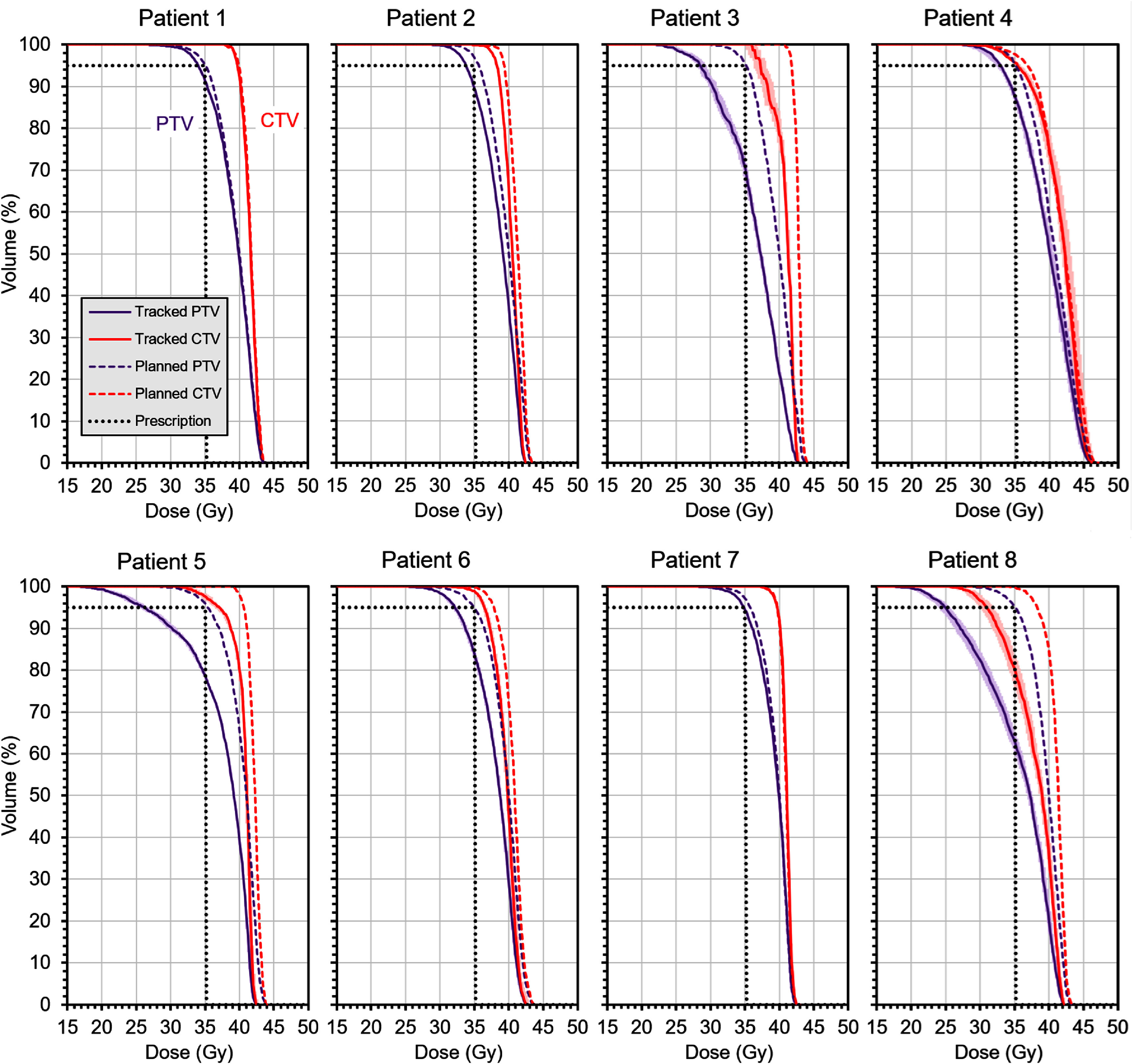
Dose-volume histograms showing the result of accumulating residual errors occurring **without** use of daily volumetric imaging. The dose for Patient 4, who is planned to 30 Gy, has been scaled up by a factor of 35/30 for consistency with the other patients. The dashed lines show the dose distribution as planned, the solid lines show the expectation value of the dose distribution with residual tracking errors. The ranges of volume resulting from 10 stochastic accumulations of dose are shown by bands of colour (most visible on patients 3 and 8 but very narrow for the other patients). The black dotted lines represent the prescription dose and volume.

The resulting dose-volume histograms for the CTV and PTV without daily volumetric imaging are shown in figure 9 after the residual tracking errors have been applied. None of the PTV structures is now covered to 95% by 35 Gy, but this is expected when taking account of tracking errors as the PTV is only a construction to ensure coverage of the CTV. Only one of the CTV structures falls substantially below the prescribed dose. This is Patient 8, who has a 3 mm PTV margin and a large median tracking error (see table 3).

**Figure 10.**
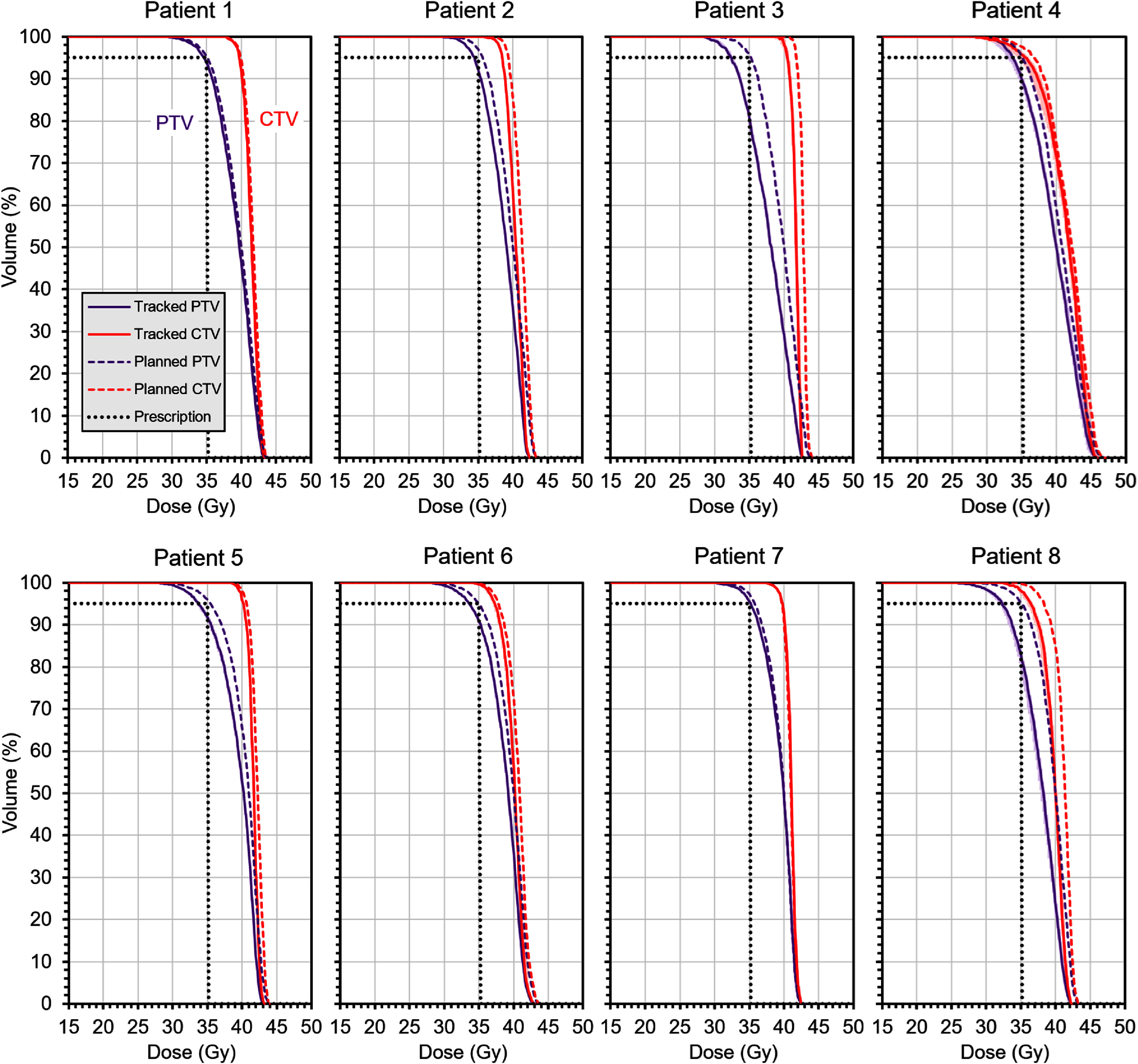
Dose-volume histograms showing the result of accumulating residual errors occurring **with** use of daily volumetric imaging. The dose for patient 4, who is planned to 30 Gy, has been scaled up by a factor of 35/30 for consistency with the other patients. The dashed lines show the dose distribution as planned, the solid lines show the expectation value of the dose distribution with residual tracking errors. The ranges of volume resulting from 10 stochastic accumulations of dose are shown by bands of colour (most visible on patient 4 but very narrow for the other patients). The black dotted lines represent the prescription dose and volume.

If daily volumetric imaging is used, the tracking performance is better, resulting in smaller residual errors. This scenario therefore gives a smaller loss in target coverage, as shown in figure 10. All of the CTVs now receive more than 35 Gy to 95% of the volume.

**Figure 11.**
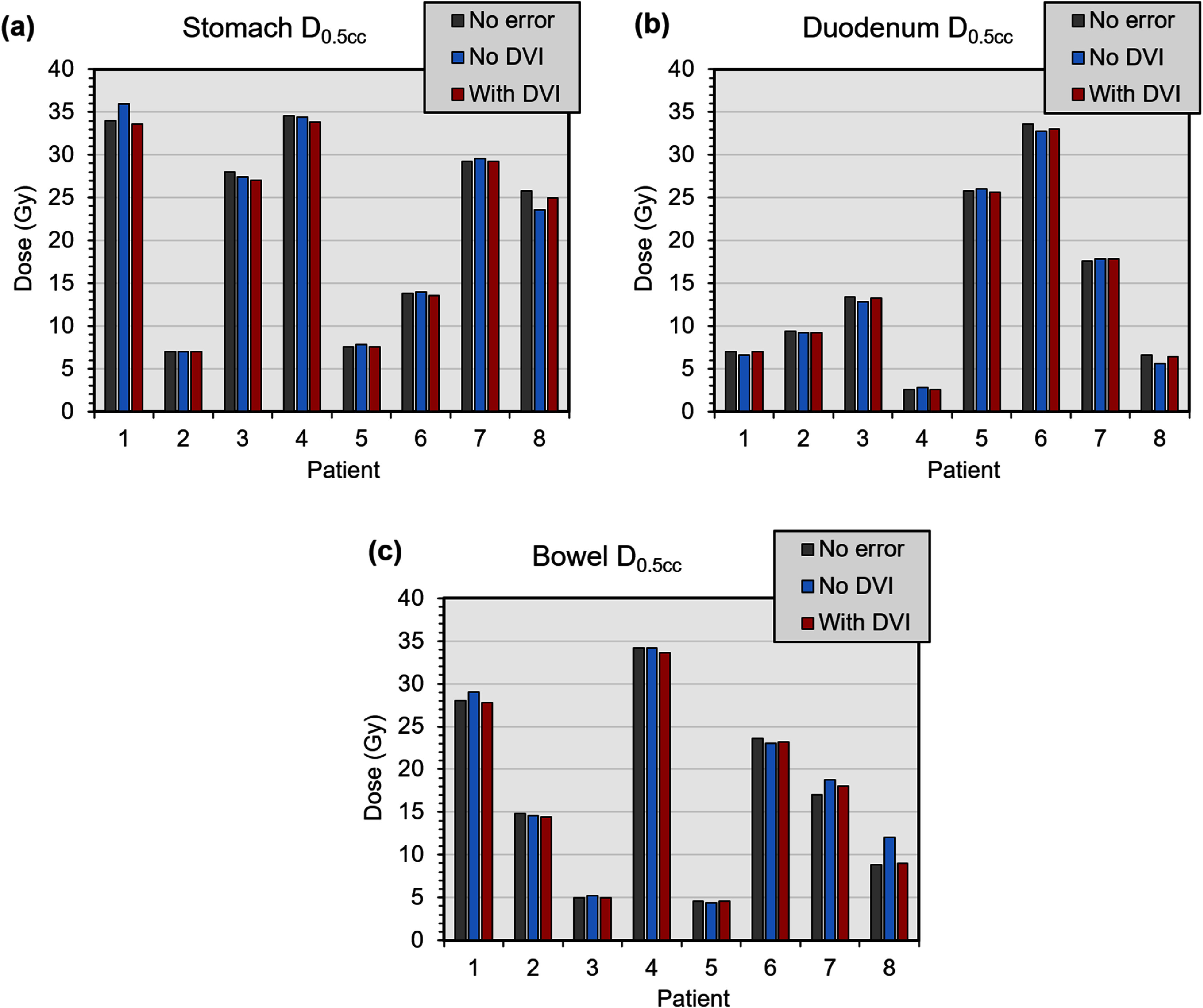
Maximum dose (*D*_0.5cc_) to (a) stomach, (b) duodenum and (c) bowel in the treatment plans delivered without error, with residual tracking error without use of daily volumetric imaging (DVI) and with residual tracking error with use of daily volumetric imaging.

The impact on the organs at risk of the residual errors is generally small, particularly with daily volumetric imaging. This is because as well as the residual shifts that move the beam closer to the organs at risk, there are also shifts that move the beam away from the organs at risk. The comparison of organ at risk dose between the normal plan, tracking without daily volumetric imaging and tracking with daily volumetric imaging is shown in figure 11.

## 4. Discussion

The results show that pancreatic tumours can be tracked without fiducial markers using the Cyberknife system to a median accuracy of 3.8 mm. The work estimates that if daily volumetric imaging is available, the accuracy can potentially be improved to 2.3 mm. The dosimetric study shows that a planning target margin of 3–5 mm, the same as with fiducial tracking, is sufficient to ensure that residual tracking error is limited to loss of coverage in the PTV, with only one patient showing the *D*_95%_ dose to the CTV to drop substantially below the prescribed dose. If daily volumetric imaging is used, the dosimetric impact of residual tracking error is negligible, which is a major motivation for implementing this type of imaging. The impact on critical structures is small, regardless of whether daily volumetric imaging is used.

Some uncertainty in pancreatic location remains after application of the motion models, particularly in the supero-inferior direction, where the motion is largest. The dispersion seen in figures 6 and 7 is the manifestation of this uncertainty. There is undoubtedly some variation in respiratory motion giving rise to the dispersion. It is also important to note that the motion models take both lateral and supero-inferior components of the registration vector at each image position and combine them to produce each component of the position vector. Thus, dispersion seen in the supero-inferior direction may be the result of uncertainty in the other directions.

There are also some practical limitations present in the study due to using a patient cohort dating from such a long time period. Although seven of the eight patients have been scanned with 4DCT, the oldest of the series, patient 8, has been scanned with a helical CT. The diaphragmatic motion in the scan and any consequent image artefacts are likely to affect the accuracy of DRR generation, and hence the accuracy of the tracking. Furthermore, the kV Cyberknife images in this patient have noticeably lower quality, which also affects the accuracy.

The use of a classical registration process before application of the motion model means that the model itself is less dependent on fitting in a large cohort of patients. The wide variation in anatomy is largely handled by the registration, and the model then further processes the result. This does mean that the registration is important in the process, and inaccuracies such as following bowel gas or overlapping vertebrae can limit the performance.

The study has been conducted in the context of Cyberknife tracking, but the results are also applicable more generally to other systems, such as the Radixact system (Accuray, Inc.) or Edge system (Varian Medical Systems, Palo Alto, CA), this latter being compared with Cyberknife by Dai *et al* (2021). The present study performs similarly to previous studies, such as that of Zhao *et al* (2019), who report a mean absolute difference between predicted position and annotated position in the order of 2.5 mm. Similarly, Nakao *et al* (2022) report a mean absolute error of 3.5 mm to 5 mm for pancreatic tracking without fiducials when including shape in the comparison. Note that both of these studies use modified DRRs to simulate treatment images, and this means that the treatment and reference images are much more similar in nature than when using DRRs as reference images and real kilovoltage images as treatment images. The inclusion of multiple patients is also difficult in this clinical site, where variation of shape and position of the target volume is large (Williams *et al* 2020). Consequently, the use of a multiple-patient model such as that applied in the present study is more difficult than using a patient-specific model, such as that of Zhao *et al* (2019). Due to these factors, the present study is considered to be much more challenging than previous studies. The study has focused on the linear models of inter- and intra-fraction motion. The trend in general is to apply whole images to a large-scale machine learning algorithm and there may be something to be gained by that approach, but the present study is able to provide results that are at least as good as the available machine learning results (Zhao *et al* 2019, Nakao *et al* 2022).

The comparison has been conducted against the clinical tracking result based on fiducial markers. The fiducial tracking algorithm used in Cyberknife is well-proven and has previously been shown to be accurate. Nevertheless, there is some uncertainty present in the clinical baseline, particularly when markers are misplaced or overlapping, and this uncertainty is likely to be in the order of ±1 mm (Akino *et al* 2018, Nano *et al* 2020, Jing *et al* 2021). There is also the possibility that the markers may migrate between planning and treatment, so that the markers do not represent the position of the pancreatic head entirely accurately. It has been shown for a similar situation in tracking liver tumours that the accuracy of true target determination reduces with increasing displacement between the fiducial markers and tumour (Seppenwoolde *et al* 2011). The estimated accuracy of ±1 mm compares with a standard deviation of error in MR-guided tracking of 0.6 mm in phantom studies and 2.1 mm in volunteers (Keiper *et al* 2020). Note, however, that in this MR-guided study, the motion traces have been offset to their centre of motion, thereby removing any systematic offset.

Cyberknife and real-time MR image guidance are both potentially valuable for pancreatic tracking. The value of MR is that the daily volumetric images have the best possible soft tissue contrast for accurate localisation of the target volume. Using real-time MR to then track the target during the treatment fraction is likely to be advantageous as it uses the same imaging modality as used for initial localisation (Ng *et al* 2023). On the other hand, determining target location currently involves observing the target in orthogonal planes, which is challenging when following complex motion (Wang *et al* 2025). Clinical implementations are currently limited to gating rather than truly tracking (Smith *et al* 2025), but multileaf collimator tracking is expected in due course (Menten *et al* 2016). In comparison, Cyberknife volumetric images are likely to have lower soft tissue contrast compared to MR. The strength of Cyberknife is its robotic delivery, in which the robot can respond quickly and smoothly to any target motion (Kilby *et al* 2020). The present study shows an accuracy of 2.3 mm with daily volumetric imaging, which is comparable to the 2.1 mm standard deviation reported by Keiper *et al* (2020) based on MR.

The magnitude of the residual tracking errors is much smaller than the motion reported by other authors during Cyberknife imaging (Knybel *et al* 2014). It is also smaller than the excursions or deformations reported in MR motion investigation (Heerkens *et al* 2014, Oar *et al* 2018, Lewis *et al* 2023) or MR-guided SABR (Alam *et al* 2022, Stanescu *et al* 2022), which indicates that the tracking method itself is able to reduce the impact of the large variations in both inter- and intra-fraction pancreatic position. The variations themselves depend on the patient immobilisation (Fontana *et al* 2016). For example, Grimbergen *et al* (2022a, 2022b) show that with abdominal compression, the intra-fraction motion can be minimised, so that the effect on dose reconstruction is minimal. More sophisticated methods, such as deforming CT scans using 4D MRI vector fields (Dolde *et al* 2018, 2019a, 2019b) have been used for investigating motion effects and accumulating dose, but for the relatively small residual errors encountered in this study after tracking, the rigid shift approach is considered to be sufficient. The impact of the residual shifts on the dose distribution delivered to the patient is mainly confined to the PTV, but there is a small impact on the organs at risk. Other authors have examined the use of a planning risk volume (PRV) for duodenum and stomach based on statistical shape modelling (Nakamura *et al* 2021), but use of a PRV is not customary at this centre for pancreatic SABR so has not been pursued.

In broad terms, the task of the inter-fraction motion model is to find the pancreas and that of the intra-fraction model is then follow the respiratory motion. It is the determination of baseline pancreas position for each fraction of treatment that remains the greatest challenge. The use of a singular value cutoff of close to unity in the inter-fraction model is necessary because it is hard to predict pancreatic location from the kV images. Nevertheless, the singular value cutoff used does preserve some of the value of the prediction model in the test patients, so that what information is available can be utilised maximally. As is well known, use of the mid-position results in the most accurate treatment in the absence of intra-fraction modelling (Hal *et al* 2020), so determination of the inter-fraction position is important. Use of daily volumetric imaging is the best means of obtaining the daily pancreatic position accurately, so this work is a major motivation to include this on the Cyberknife system. Such imaging has already been included in an in-house context (Papalazarou *et al* 2017) and work is in progress at Accuray with a view to providing this facility as part of the commercial device.

This study has estimated the accuracy of tracking when the inter-fraction mean position is perfectly known. In reality, there is likely to be some uncertainty in this position due to limitations of soft tissue contrast, registration uncertainty and physical shift errors. The latter are shown by Papalazarou *et al* (2017) to be within 1 mm, regardless of whether the couch or robot is shifted. However, accuracy of the pancreas localisation and registration is expected to increase this uncertainty.

The study by Nano *et al* (2020) estimates the dose to the patient from the planning CT scan to be in the order of 15–30 mGy, while that of each pair of orthogonal tracking images is approximately 1–2.5 mGy. Given that there are typically 500 tracking images during the course of a pancreas Cyberknife treatment, the total dose from imaging is in the order of 1 Gy. This is the case whether fiducial markers are used for treatment or not, so the proposed approach does not increase the imaging dose compared to current practice. However, using daily volumetric imaging would increase the patient dose by approximately 100 mGy. This may be considered an acceptable increase given the benefit of avoiding implantation of fiducial markers. The total imaging dose, even with daily volumetric imaging, is approximately 3% of the prescribed dose, which is within the guidelines suggested by Ding *et al* (2018b).

Further improvements in quality of tracking may be made by increasing the accuracy of the image registration step and by using a more sophisticated motion model. Dual-energy imaging may also allow the visualisation of bony and soft tissue anatomy, allowing differentiation between the overall patient position and the pancreatic position. While accuracy has been the focus of this study, the speed of the marker-less tracking algorithm has also been a relevant factor. Application in practice requires that the pancreatic position can be determined in less than about one second. In the Cyberknife system, the Synchrony tracking algorithm handles respiratory motion throughout the treatment, but the position results obtained from the imaging panels must be sufficiently fast to build the initial respiratory model. Further optimisation of the tracking speed is possible, either by extending the multi-threaded approach already present in the software, or by using graphics processing units.

## Data Availability

The data cannot be made publicly available upon publication because they contain sensitive personal information. The data that support the findings of this study are available upon reasonable request from the authors.

## References

[pmbae6229bib1] Akino Y, Sumida I, Shiomi H, Higashinaka N, Murashima Y, Hayashida M, Mabuchi N, Ogawa K (2018). Evaluation of the accuracy of the CyberKnife Synchrony^TM^ respiratory tracking system using a plastic scintillator. Med. Phys..

[pmbae6229bib2] Alam S, Veeraraghavan H, Tringale K, Amoateng E, Subashi E, Wu A J, Crane C H, Tyagi N (2022). Inter- and intrafraction motion assessment and accumulated dose quantification of upper gastrointestinal organs during magnetic resonance-guided ablative radiation therapy of pancreas patients. Phys. Imaging Radiat. Oncol..

[pmbae6229bib3] Asmerom G (2016). The design and physical characterization of a multileaf collimator for robotic radiosurgery. Biomed. Phys. Eng. Express.

[pmbae6229bib4] Bedford J L (2002). Speed versus accuracy in a fast convolution photon dose calculation for conformal radiotherapy. Phys. Med. Biol..

[pmbae6229bib5] Bedford J L (2024). Inverse planning of lung radiotherapy with photon and proton beams using a discrete ordinates Boltzmann solver. Phys. Med. Biol..

[pmbae6229bib6] Bedford J L, Nill S, Oelfke U (2020). Dosimetric accuracy of delivering SBRT using dynamic arcs on Cyberknife. Med. Phys..

[pmbae6229bib7] Colvill E (2016). A dosimetric comparison of real-time adaptive and non-adaptive radiotherapy: a multi-institutional study encompassing robotic, gimbaled, multileaf collimator and couch tracking. Radiat. Oncol..

[pmbae6229bib8] Czolbe S, Pegios P, Krause O, Feragen A (2023). Semantic similarity metrics for image registration. Med. Image Anal..

[pmbae6229bib9] Dai Z, Ma L, Cao T, Zhu L, Zhao M, Li N (2021). Dosimetric and radiobiological comparison of treatment plans between CyberKnife and EDGE in stereotactic body radiotherapy for pancreatic cancer. Sci. Rep..

[pmbae6229bib10] Deng Z (2019). A post-processing method based on interphase motion correction and averaging to improve image quality of 4D magnetic resonance imaging: a clinical feasibility study. Br. J. Radiol..

[pmbae6229bib11] Deng Z, Yang W, Pang J, Bi X, Tuli R, Li D, Fan Z (2017). Improved vessel–tissue contrast and image quality in 3D radial sampling-based 4D-MRI. J. Appl. Clin. Med. Phys..

[pmbae6229bib12] Dhont J, Verellen D, Mollaert I, Vanreusel V, Vandemeulebroucke J (2020). RealDRR—Rendering of realistic digitally reconstructed radiographs using locally trained image-to-image translation. Radiat. Oncol..

[pmbae6229bib13] Ding C, Saw C B, Timmerman R D (2018a). Cyberknife stereotactic radiosurgery and radiation therapy treatment planning system. Med. Dosim..

[pmbae6229bib14] Ding G X, Alaei P, Curran B, Flynn R, Gossman M, Mackie T R, Miften M, Morin R, Xu X G, Zhu T C (2018b). Image guidance doses delivered during radiotherapy: quantification, management, and reduction: report of the AAPM therapy physics committee task group 180. Med. Phys..

[pmbae6229bib15] Dolde K, Naumann P, Dávid C, Gnirs R, Kachelriess M, Lomax A J, Saito N, Weber D C, Pfaffenberger A, Zhang Y (2018). 4D dose calculation for pencil beam scanning proton therapy of pancreatic cancer using repeated 4DMRI datasets. Phys. Med. Biol..

[pmbae6229bib16] Dolde K, Naumann P, Dávid C, Kachelriess M, Lomax A J, Weber D C, Saito N, Burigo L N, Pfaffenberger A, Zhang Y (2019a). Comparing the effectiveness and efficiency of various gating approaches for PBS proton therapy of pancreatic cancer using 4D-MRI datasets. Phys. Med. Biol..

[pmbae6229bib17] Dolde K, Zhang Y, Chaudhri N, Dávid C, Kachelrieß M, Lomax A J, Naumann P, Saito N, Weber D C, Pfaffenberger A (2019b). 4DMRI-based investigation on the interplay effect for pencil beam scanning proton therapy of pancreatic cancer patients. Radiat. Oncol..

[pmbae6229bib18] Ferris W S, Kissick M W, Bayouth J E, Culberson W S, Smilowitz J B (2020). Evaluation of radixact motion synchrony for 3D respiratory motion: modeling accuracy and dosimetric fidelity. J. Appl. Clin. Med. Phys..

[pmbae6229bib19] Fontana G (2016). MRI quantification of pancreas motion as a function of patient setup for particle therapy—a preliminary study. J. Appl. Clin. Med. Phys..

[pmbae6229bib20] Fransson S, Tilly D, Ahnesjö A, Nyholm T, Strand R (2021). Intrafractional motion models based on principal components in Magnetic Resonance guided prostate radiotherapy. Phys. Imaging Radiat. Oncol..

[pmbae6229bib21] Grimbergen G, Eijkelenkamp H, Heerkens H D, Raaymakers B W, Intven M P W, Meijer G J (2022a). Intrafraction pancreatic tumor motion patterns during ungated magnetic resonance guided radiotherapy with an abdominal corset. Phys. Imaging Radiat. Oncol..

[pmbae6229bib22] Grimbergen G, Eijkelenkamp H, Heerkens H D, Raaymakers B W, Intven M P W, Meijer G J (2022b). Dosimetric impact of intrafraction motion under abdominal compression during MR-guided SBRT for (peri-) pancreatic tumors. Phys. Med. Biol..

[pmbae6229bib23] Hal W A, Straza M W, Chen X, Mickevicius N, Erickson B, Schultz C, Awan M, Ahunbay E, Li X A, Paulson E S (2020). Initial clinical experience of stereotactic body radiation therapy (SBRT) for liver metastases, primary liver malignancy, and pancreatic cancer with 4D-MRI based online adaptation and real-time MRI monitoring using a 1.5 Tesla MR-Linac. PLoS One.

[pmbae6229bib24] Hall W A (2021). Magnetic resonance guided radiation therapy for pancreatic adenocarcinoma, advantages, challenges, current approaches, and future directions. Front. Oncol..

[pmbae6229bib25] Heerkens H D, van Vulpen M, van den Berg C A T, Tijssen R H N, Crijns S P M, Molenaar I Q, van Santvoort H C, Reerink O, Meijer G J (2014). MRI-based tumor motion characterization and gating schemes for radiation therapy of pancreatic cancer. Radiat. Oncol..

[pmbae6229bib26] International Commission on Radiation Units and Measurements (1992). ICRU Report 46: Photon, Electron, Proton and Neutron Interaction Data for Body Tissues.

[pmbae6229bib27] Jing S, Jiang C, Ji X, Qiu X, Li J, Sun X, Zhu X (2021). Study on motion management of pancreatic cancer treated by CyberKnife. Front. Oncol..

[pmbae6229bib28] Keiper T D, Tai A, Chen X, Paulson E, Lathuilière F, Bériault S, Hébert F, Cooper D T, Lachaine M, Li X A (2020). Feasibility of real-time motion tracking using cine MRI during MR-guided radiation therapy for abdominal targets. Med. Phys..

[pmbae6229bib29] Kilby W, Naylor M, Dooley J R, Maurer Jr C R, Sayeh S, Abedin-Nasab M H (2020). A technical overview of the Cyberknife system. Handbook of Robotic and Image-Guided Surgery.

[pmbae6229bib30] Knybel L, Cvek J, Otahal B, Jonszta T, Molenda L, Czerny D, Skacelikova E, Rybar M, Dvorak P, Feltl D (2014). The analysis of respiration-induced pancreatic tumor motion based on reference measurement. Radiat. Oncol..

[pmbae6229bib31] Kumar S, Panda D K, Sharma P (2025). A comparison of tumor tracking accuracy using real-rime adaptive motion management on helical versus robotic radiotherapy platforms: an interdisciplinary study. Cureus.

[pmbae6229bib32] Lewis B, Guta A, Shin J, Ji Z, Kim J S, Kim T (2023). Evaluating motion of pancreatic tumors and anatomical surrogates using cine MRI in 0.35T MRgRT under free breathing conditions. J. Appl. Clin. Med. Phys..

[pmbae6229bib33] Menten M J, Fast M F, Nill S, Kamerling C P, McDonald F, Oelfke U (2016). Lung stereotactic body radiotherapy with an MR-linac—quantifying the impact of the magnetic field and real-time tumor tracking. Radiat. Oncol..

[pmbae6229bib34] Mylonas A, Booth J, Nguyen D T (2021). A review of artificial intelligence applications for motion tracking in radiotherapy. J. Med. Imaging Radiat. Oncol..

[pmbae6229bib35] Nakamura M, Nakao M, Mukumoto N, Ashida R, Hirashima H, Yoshimura M, Mizowaki T (2021). Statistical shape model-based planning organ-at-risk volume: application to pancreatic cancer patients. Phys. Med. Biol..

[pmbae6229bib36] Nakao M, Nakamura M, Matsuda T (2022). Image-to-graph convolutional network for 2D/3D deformable model registration of low-contrast organs. IEEE Trans. Med. Imaging.

[pmbae6229bib37] Nano T F, Capaldi D P I, Yeung T, Chuang C F, Wang L, Descovich M (2020). Technical note: performance of CyberKnife tracking using low-dose CT and kV imaging. Med. Phys..

[pmbae6229bib38] Ng J, Gregucci F, Pennell R T, Nagar H, Golden E B, Knisely J P S, Sanfilippo N J, Formenti S C (2023). MRI-LINAC: a transformative technology in radiation oncology. Front. Oncol..

[pmbae6229bib39] Oar A, Liney G, Rai R, Deshpande S, Pan L, Johnston M, Jameson M, Kumar S, Lee M (2018). Comparison of four dimensional computed tomography and magnetic resonance imaging in abdominal radiotherapy planning. Phys. Imaging Radiat. Oncol..

[pmbae6229bib40] Papalazarou C, Klop G J, Milder M T W, Marijnissen J P A, Gupta V, Heijmen B J M, Nuyttens J J M E, Hoogeman M S (2017). CyberKnife with integrated CT-on-rails: system description and first clinical application for pancreas SBRT. Med. Phys..

[pmbae6229bib41] Press W H, Flannery B P, Teukolsky S A, Vetterling W T (1989). Numerical Recipes in Pascal.

[pmbae6229bib42] Royal College of Radiologists (2019). Stereotactic Ablative Body Radiation Therapy (SABR): a resource. https://www.sabr.org.uk/wp-content/uploads/2019/04/SABRconsortium-guidelines-2019-v6.1.0.pdf.

[pmbae6229bib43] Salari E, Wang J, Wynne J F, Chang C-W, Wu Y, Yang X (2024). Artificial intelligence-based motion tracking in cancer radiotherapy: a review. J. Appl. Clin. Med. Phys..

[pmbae6229bib44] Seppenwoolde Y, Wunderink W, Wunderink-van Veen S R, Storchi P, Méndez Romero A, Heijmen B J M (2011). Treatment precision of image-guided liver SBRT using implanted fiducial markers depends on marker–tumour distance. Phys. Med. Biol..

[pmbae6229bib45] Shouman M A (2024). Stereotactic body radiotherapy for pancreatic cancer—a systematic review of prospective data. Clin. Translational. Radiat. Oncol..

[pmbae6229bib46] Smith B, Allen B, Rusu S, St-Aubin J, Hyer D (2025). Real-time tracking of lung tumors using a 1.5T Elekta Unity MR-Linac: first clinical experiences. Front. Oncol..

[pmbae6229bib47] Song Y, Yuan Z, Li F, Dong Y, Zhuang H, Wang J, Chen H, Wang P (2015). Analysis of clinical efficacy of CyberKnife® treatment for locally advanced pancreatic cancer. OncoTargets Ther..

[pmbae6229bib48] Stanescu T, Shessel A, Carpino-Rocca C, Taylor E, Semeniuk O, Li W, Barry A, Lukovic J, Dawson L, Hosni A (2022). MRI-guided online adaptive stereotactic body radiation therapy of liver and pancreas tumors on an MR-linac system. Cancers.

[pmbae6229bib49] Stemkens B, Tijssen R H N, de Senneville B D, Heerkens H D, van Vulpen M, Lagendijk J J W, van den Berg C A T (2015). Optimizing 4-dimensional magnetic resonance imaging data sampling for respiratory motion analysis of pancreatic tumors. Int. J. Radiat. Oncol. Biol. Phys..

[pmbae6229bib50] Studholme C, Hill D L G, Hawkes D J (1999). An overlap invariant entropy measure of 3D medical image alignment. Pattern Recognit..

[pmbae6229bib51] Teuwen J, Gouw Z A R, Sonke J-J (2022). Artificial intelligence for image registration in radiation oncology. Semin. Radiat. Oncol..

[pmbae6229bib52] Wang H, Brock K K (2014). Similarity metrics. Image Processing in Radiation Therapy.

[pmbae6229bib53] Wang Y, Lombardo E, Wang J, Fan Y, Zhao Y, Corradini S, Belka C, Riboldi M, Kurz C, Landry G (2025). Real-time target localization on 1.5 T magnetic resonance imaging linac orthogonal cine images using transfer learning. Phys. Imaging Radiat. Oncol..

[pmbae6229bib54] Williams J M, Hilmes M A, Archer B, Dulaney A, Du L, Kang H, Russell W E, Powers A C, Moore D J, Virostko J (2020). Repeatability and reproducibility of pancreas volume measurements using MRI. Sci. Rep..

[pmbae6229bib55] Yang W, Fan Z, Deng Z, Pang J, Bi X, Fraass B A, Sandler H, Li D, Tuli R (2018). Novel 4D-MRI of tumor infiltrating vasculature: characterizing tumor and vessel volume motion for selective boost volume definition in pancreatic radiotherapy. Radiat. Oncol..

[pmbae6229bib56] Yang W, Fan Z, Tuli R, Deng Z, Pang J, Wachsman A, Reznik R, Sandler H, Li D, Fraass B A (2015). Four-dimensional magnetic resonance imaging with 3-dimensional radial sampling and self-gating-based k-space sorting: early clinical experience on pancreatic cancer patients. Int. J. Radiat. Oncol. Biol. Phys..

[pmbae6229bib57] Zhao W, Shen L, Han B, Yang Y, Cheng K, Toesca D A S, Koong A C, Chang D T, Xing L (2019). Markerless pancreatic tumor target localization enabled by deep learning. Int. J. Radiat. Oncol. Biol. Phys..

[pmbae6229bib58] Zhao W, Shen L, Islam M, Qin W, Zhang Z, Liang X, Zhang G, Xu S, Li X (2021). Artificial intelligence in image-guided radiotherapy: a review of treatment target localization. Quant. Imaging Med. Surg..

[pmbae6229bib59] Zhou D, Nakamura M, Mukumoto N, Yoshimura M, Mizowaki T (2022). Development of a deep learning-based patient-specific target contour prediction model for markerless tumor positioning. Med. Phys..

